# Supplementary material for: Multi-scale parameterization of neural rhythmicity with lagged Hilbert autocoherence
Source: Imaging Neurosci (Camb). 2025 Nov 10;3:IMAG.a.993. doi: 10.1162/IMAG.a.993 (PMC12603660; doi:10.1162/IMAG.a.993)
Supplement: Supplementary Material [file IMAG.a.993_supp.pdf]

Supplementary Figures

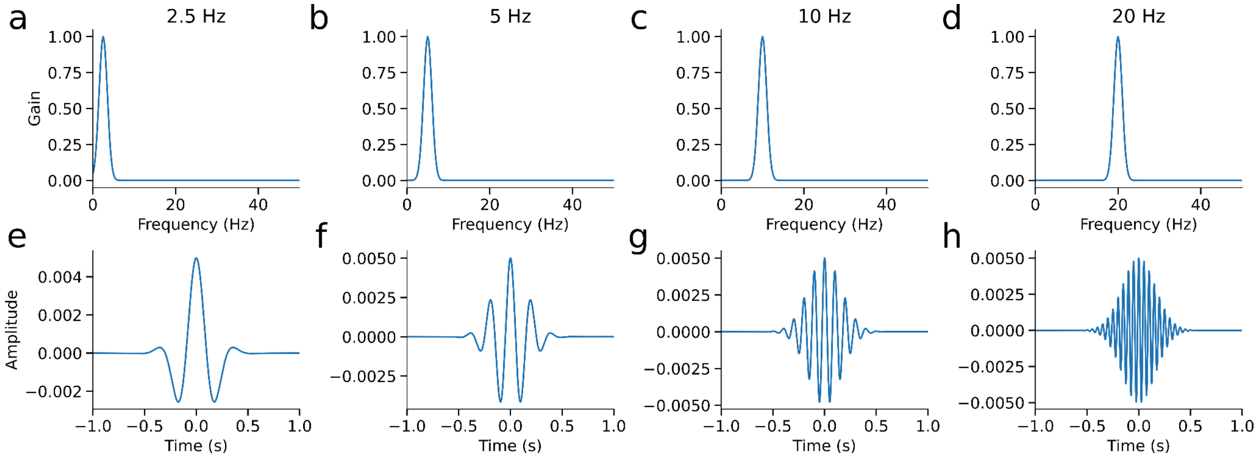

Figure S1. Frequency and impulse responses of Gaussian filters used for frequency-domain bandpass filtering. a-d) Frequency responses of Gaussian kernels centered at 2.5, 5, 10, and 20 Hz, respectively, each with fixed spectral resolution (σ = df/2, where df = 2 Hz). e-h) Corresponding time-domain impulse responses, obtained by inverse Fourier transform of the Gaussian frequency kernels.

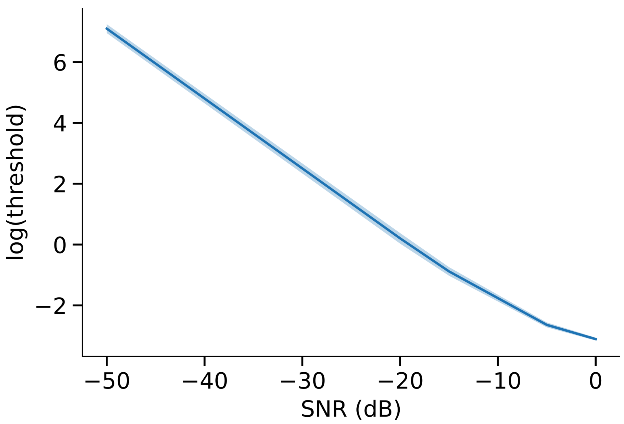

Figure S2. Relationship between signal-to-noise ratio (SNR) and the surrogate threshold for the joint amplitude normalization factor used in lagged Hilbert autocoherence (LHaC). Simulated signals consisted of 15 Hz bursts embedded in pink noise at varying SNR levels. For each SNR, the 95th percentile of the joint amplitude normalization factor was computed from 1000 phase-shuffled surrogates. Shown are the mean and standard deviation of the log-transformed thresholds across trials. As SNR increases, the surrogate threshold decreases, reflecting reduced amplitude normalization in signals with stronger oscillatory components.

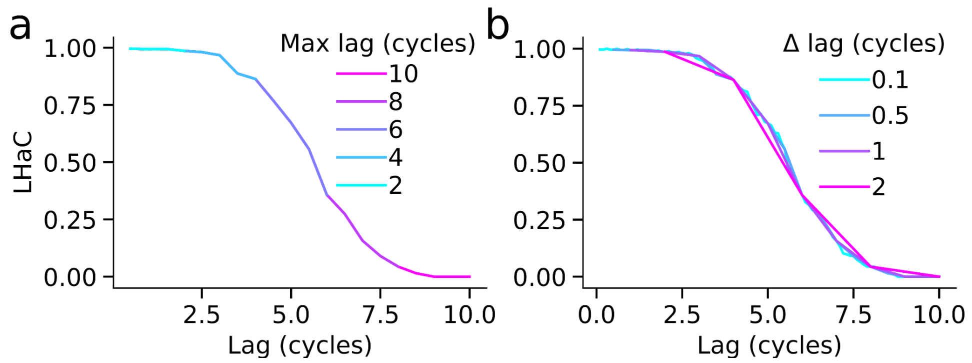

Figure S3. Sensitivity of lagged Hilbert autocoherence (LHaC) to lag sampling parameters. Simulated data contained transient 15 Hz bursts embedded in 1/f noise. a) Mean LHaC at 15 Hz as a function of lag duration for five maximum lag values (2-10 cycles), with lag increments fixed at 0.5 cycles. Increasing the maximum lag reveals the timescale over which coherence persists. b) Mean LHaC at 15 Hz for different lag sampling intervals (Δ lag = 0.1-2 cycles), with the maximum lag fixed at 10 cycles. While finer increments yield smoother LHaC curves, the overall coherence profile remains stable across settings, indicating that LHaC is robust to reasonable variation in lag sampling parameters.

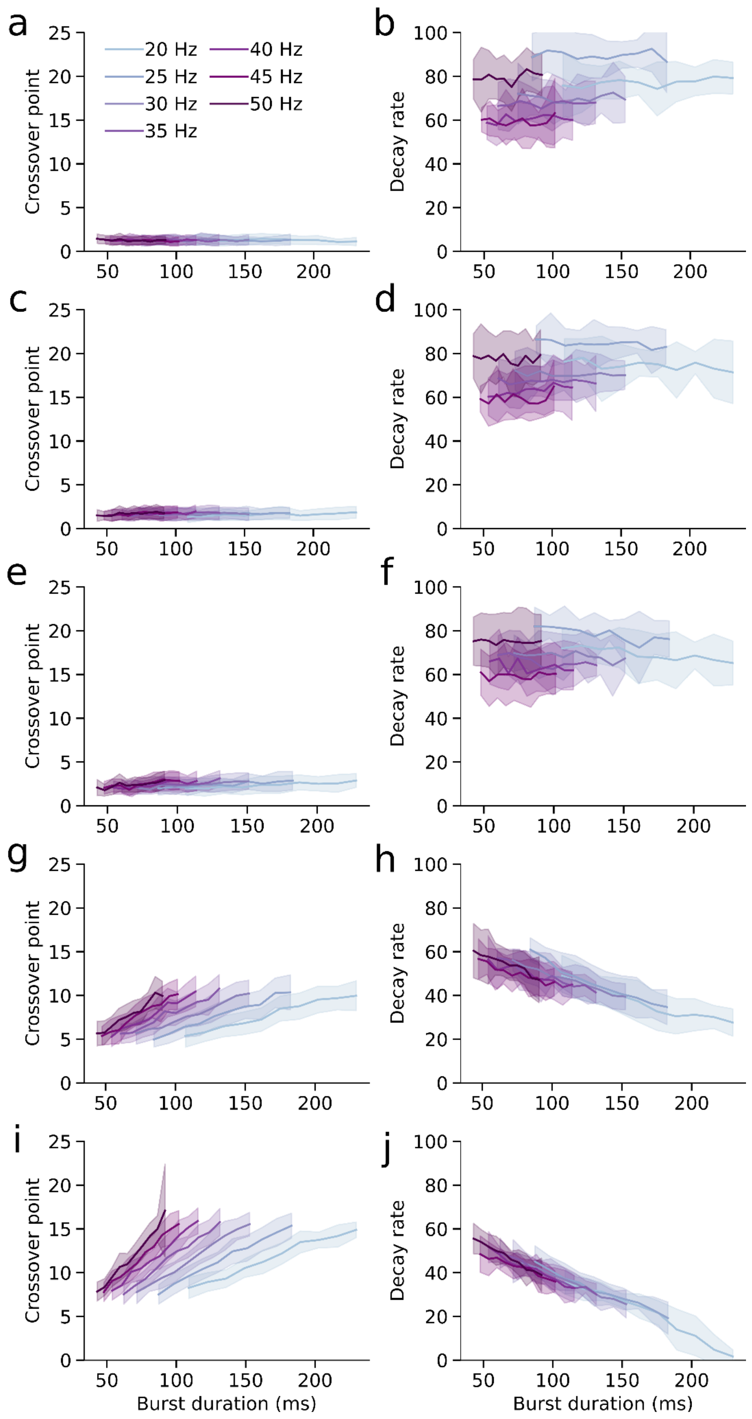

Figure S4. Lagged Hilbert autocoherence (LHaC) reflects burst duration across signal-to-noise ratios (SNRs). a-j) At each SNR level (-50, -20, -15, -5, and 0 dB, top to bottom), we evaluated the relationship between burst duration in milliseconds (x-axis) and the fitted parameters of an inverse sigmoid model applied to LHaC: the crossover point (left column) and decay rate (right column). Each line represents a different simulated frequency (20-50 Hz), with shaded areas indicating ±1 SD across trials. As SNR increases, both the crossover point

and decay rate show progressively stronger and more monotonic relationships with burst duration across frequencies.

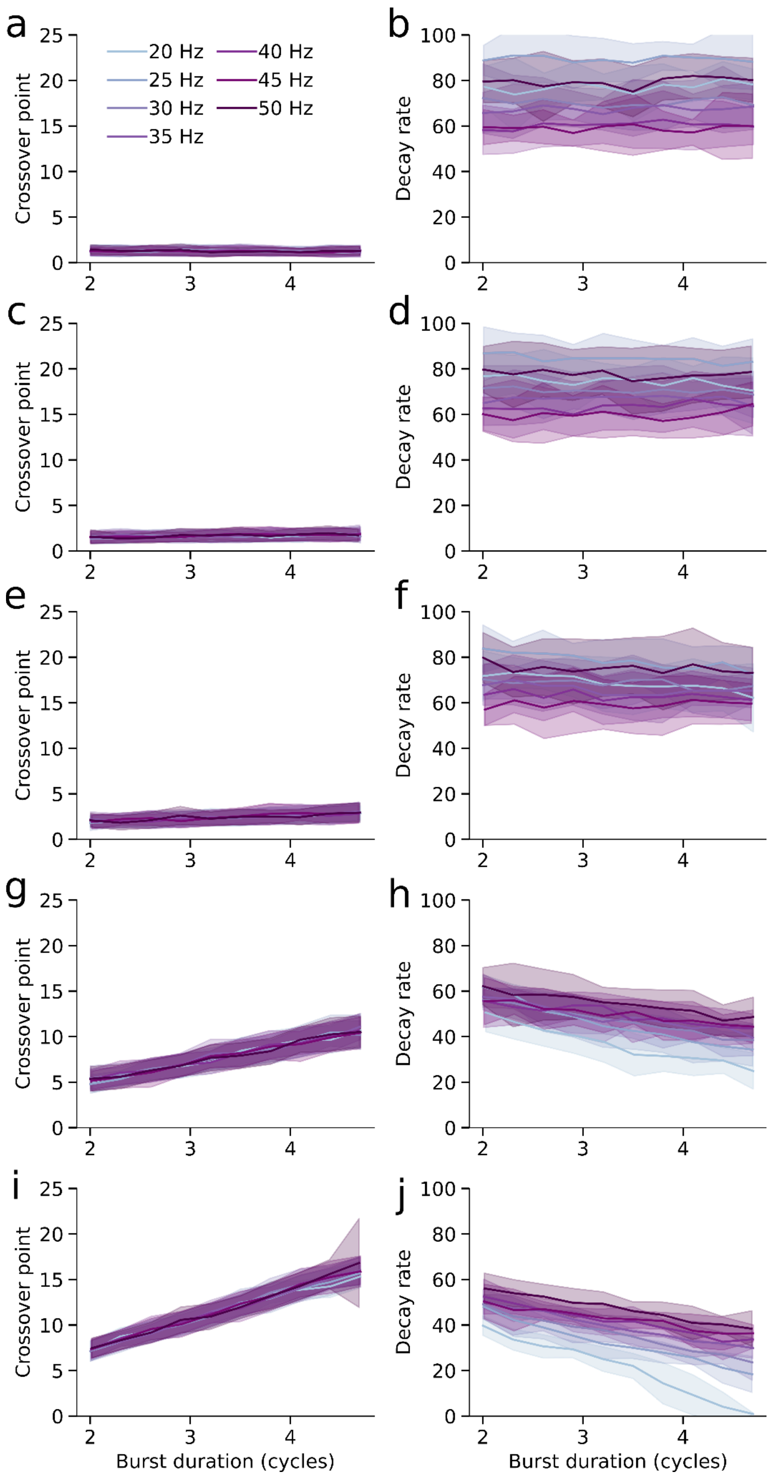

Figure S5. Lagged Hilbert autocoherence (LHaC) reflects burst duration in cycles across signal-to-noise ratios (SNRs). a-j) As in Figure S4, we examined the relationship between burst duration and LHaC parameters, but here duration is expressed in cycles rather than milliseconds. Each subplot pair shows the crossover point (left) and decay rate (right) of the

inverse sigmoid fit to LHaC, plotted as a function of burst duration in cycles at an SNR of -50, -20, -15, -5, and 0 dB (top to bottom).

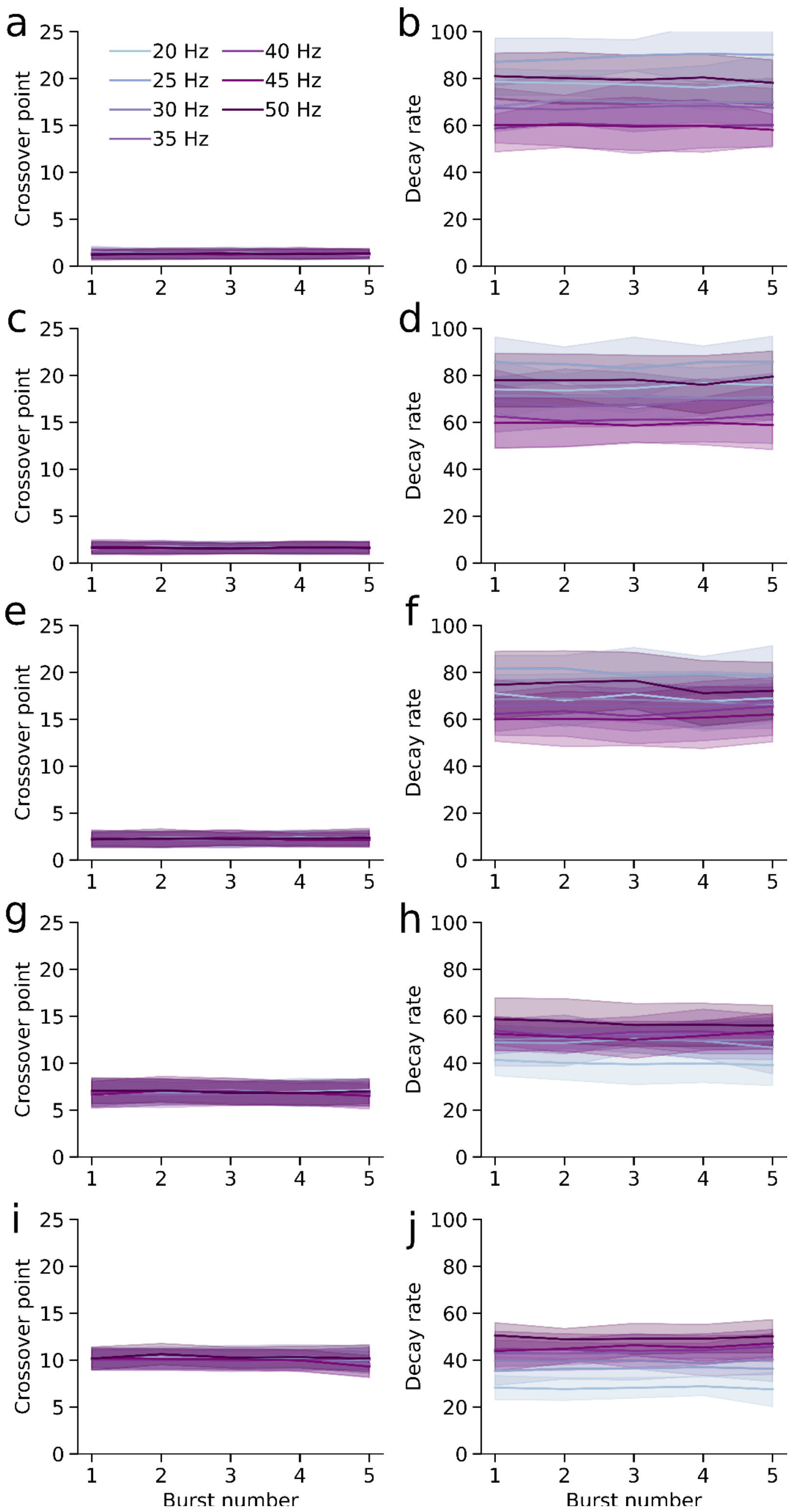

Figure S6. Lagged Hilbert autocoherence (LHaC) is invariant to burst count across signal-to-noise ratios (SNRs). a-j) As in Figure S4, we examined the relationship between burst number and LHaC parameters. Each subplot pair shows the crossover point (left) and

decay rate (right) of the inverse sigmoid fit to LHaC, plotted as a function of burst number at an SNR of -50, -20, -15, -5, and 0 dB (top to bottom).
